# Translating the foundational model of anatomy into french using knowledge-based and lexical methods

**DOI:** 10.1186/1472-6947-11-65

**Published:** 2011-10-26

**Authors:** Tayeb Merabti, Lina F Soualmia, Julien Grosjean, Olivier Palombi, Jean-Michel Müller, Stéfan J Darmoni

**Affiliations:** 1 CISMeF, Rouen University Hospital, Normandy & TIBS, LITIS EA 4108, Institute of Biomedical Research, Rouen, France; 2 LIM&Bio EA 3969, Paris X I I I University, Sorbonne Paris Cité, Bobigny, France; 3 Laboratory of Anatomy of Grenoble University, Grenoble, France

## Abstract

**Background:**

The Foundational Model of Anatomy (FMA) is the reference ontology regarding human anatomy. FMA vocabulary was integrated into the Health Multi Terminological Portal (HMTP) developed by CISMeF based on the CISMeF Information System which also includes 26 other terminologies and controlled vocabularies, mainly in French. However, FMA is primarily in English. In this context, the translation of FMA English terms into French could also be useful for searching and indexing French anatomy resources. Various studies have investigated automatic methods to assist the translation of medical terminologies or create multilingual medical vocabularies. The goal of this study was to facilitate the translation of FMA vocabulary into French.

**Methods:**

We compare two types of approaches to translate the FMA terms into French. The first one is UMLS-based on the conceptual information of the UMLS metathesaurus. The second method is lexically-based on several Natural Language Processing (NLP) tools.

**Results:**

The UMLS-based approach produced a translation of 3,661 FMA terms into French whereas the lexical approach produced a translation of 3,129 FMA terms into French. A qualitative evaluation was made on 100 FMA terms translated by each method. For the UMLS-based approach, among the 100 translations, 52% were manually rated as "very good" and only 7% translations as "bad". For the lexical approach, among the 100 translations, 47% were rated as "very good" and 20% translations as "bad".

**Conclusions:**

Overall, a low rate of translations were demonstrated by the two methods. The two approaches permitted us to semi-automatically translate 3,776 FMA terms from English into French, this was to added to the existing 10,844 French FMA terms in the HMTP (4,436 FMA French terms and 6,408 FMA terms manually translated).

## Introduction

Biomedical terminologies and ontologies have proliferated during the past decade. Due to this proliferation, health care systems use different biomedical terminologies. Anatomical knowledge is central to biomedical applications, including automated coding of Electronic Health Records, free-text indexing and information retrieval. Various representations of anatomy have been developed (*e.g*. Adult Mouse Anatomical Dictionary, the Foundational Model of Anatomy (FMA), ... etc), but their coverage varies according to the language. The French language, while being fairly well represented in several medical terminologies and controlled vocabularies (such as MeSH, SNOMED International and ICD-10) could still benefit from the addition of new terms based on vocabularies associated to the lexicon of the FMA ontology or the SNOMED CT.

The catalogue of online health resources in French (CISMeF) [1] is an example of an application which is based on French-language medical terminology resources. CISMeF was originally indexed on the basis of only one medical thesaurus: the Medical Subject Headings (MeSH). Since 2005, we have decided to use the main health terminologies available in French for automatic indexing and information retrieval [2]. In this context, the addition of new French terminologies would be particularly useful, for instance through the translation of some or the many existing English language standards. The FMA vocabulary is a good example of this type of terminology not translated (in its full version) into French. The French translation of FMA terms available in English will be useful to index and to search anatomical resources through the CISMeF.

In this study, we propose two approaches to automatically translate the FMA from English into French: a knowledge-based approach that mainly relies on the Unified Medical Language System resources (UMLS^®^) [3], and Natural Language Processing (NLP) approach using the Multi-Terminolgical CISMeF Information System (CISMeF_IS) [2] that contains 27 terminologies (see Table 1). The main objective of this paper aims at comparing the two approaches (UMLS-based and lexical) to determine the strengths and weaknesses of each approach.

**Table 1:** List of terminologies included in the HMTP

Terminology	HMTP	UMLS
CCAM	Included (Fr and En)	

CISMeF	Included (Fr and En)	

CLADIMED	Included (Fr)	Included (En)

Codes used for drugs	Included (Fr and En)	

DRC	Included (Fr and En)	

FMA	Included (Fr and En)	Included (En)

ICD10	Included (Fr and En)	Included (En)

IDIT	Included (Fr)	

IUPAC	Included (Fr and En)	

LOINC	Included (Partially translated Fr, En)	Included (En)

LPP	Included (Fr)	

MedDRA	Included (Fr and En)	Included (Fr and En)

MEDLINEPlus	Included (Fr and En)	Included (En)

MeSH	Included (Fr and En)	Included (Fr and En)

NCCMERP	Included (En)	

ORPHANET	Included (Fr and En)	

PSIP Taxo.	Included (En)	

SNOMED CT	Included (En)	Included (En)

SNOMED International	Included (Fr and En)	Included (En)

TUV	Included (Fr and En)	

UNIT	Included (Fr and En)	

VCM	Included (Fr)	

WHO-ART	Included (Fr and En)	Included (Fr and En)

WHO-ATC	Included (Fr and En)	

WHO-ICF	Included (Fr and En)	Included (En)

WHO-ICPC2	Included (Fr and En)	Included (Fr and En)

WHO-ICPS	Included (Fr and En)	

## Background

Various studies have investigated automatic methods to assist the translation of medical terminologies or to create multilingual medical vocabularies. Some of these methods use rewriting rules to translate biomedical terms: in [4] the authors proposed a method to translate biomedical terms from Portuguese into Spanish. This method is also applied for information retrieval [5]. However, as stated in [6], rules used are hand-coded, which renders this approach not transferable to other languages and domains. The method proposed in [6] relies on an automatic process able to infer rewriting rules from examples. These examples represent a list of paired terms in two studied languages (pair terms from Masson medical dictionary and from the UMLS metathesaurus). In [6] the author has presented an automatic method that relies on machine learning technique. It can infer transducers from examples of bilingual word pairs without any additional resource or knowledge. In contrast, some methods use existing terminological resources to translate medical terminologies: in our previous work [7] we proposed a semantics-based method to assist the translation of SNOMED CT into French. We also used the UMLS Metathesaurus restricted to four French terminologies. Recently, we combined a UMLS-based approach and a corpus-based approach to translate MEDLINEPlus^® ^Topics from English into French [8]. This UMLS-based approach was used in BabelMeSH [9] to automatically translate a query from French, Spanish and Portuguese into English to allow querying MEDLINE^® ^via PubMed^® ^with such languages.

In order to create a multilingual dictionary, the authors in [10] mapped monolingual medical lexicons using morphological decomposition. In [11], the authors proposed a method that uses various parallel terminologies to build an English-Swedish medical dictionary.

Other types of methods are based on text corpora to acquire translations of medical terms [12-16]. These multilingual text corpora can be parallel: i.e. texts in different languages that are translations of each other such as those used to match English UMLS terms with their German translations [12] or to find French translations of MeSH terms [13]. The multilingual text corpora can be comparable: i.e. texts addressing the same general topic in different languages, to search for French translations of medical terms [14], to extend the German version of the MeSH [15] or to build a Japanese-French terminology [16]. Approaches developed in our study are mapping methods developed beforehand regarding the creation of mappings between terms from different terminologies [17].

## Material

### The Unified Medical Language System (UMLS)

The UMLS [3] is a repository of biomedical vocabularies developed by the US National Library of Medicine. It integrates over two million concepts (2,200,159 in the 2010AB version) from 154 biomedical vocabularies. The UMLS is made up of three main knowledge components, but, for our purpose, we retained the Metathesaurus. It is a very large, multi-purpose, and multilingual vocabulary database that contains information about biomedical and health-related concepts, their various names, and the relationships between them. It is built from the electronic versions of many different thesauri, classifications, code sets, and lists of controlled terms used in patient care, health services billing, public health statistics, biomedical literature indexing and cataloging, and health services research. Within the Metathesaurus we will use more specifically the MRCONSO table, which lists all the concepts that are incorporated in the UMLS. Each concept has a Concept Unique Identifier (CUI) in this table. This means that the same concept that may appear in various terminologies, perhaps with various names and synonyms, has a unique entry in the Metathesaurus. Thus, the concept identifier allows to link together the different terminologies included in the UMLS.

According to the 154 biomedical vocabularies in the UMLS, there are only six French terminologies included: the MeSH^® ^thesaurus [18], the International Classification of Diseases (ICD10) [19], the Systematized NOmenclature of MEDicine (SNOMED Int.) [20], the World Health Organization Adverse Reaction Terminology (WHO-ART) [21], the WHO International Classification of Primary Care(ICPC2) [22] and the Medical Dictionary of Regulatory Activities (MedDRA) [23]. Nevertheless, only four (4) terminologies are included with their French version in UMLS Metathesaurus (MeSH, WHO-ART, WHO-ICPC2 and MedDRA). However, several translations had already been added, such as MEDLINEPlus [8] and partially the Logical Observation Identifiers Names and Codes (LOINC).

### CISMeF Information System (CISMeF_IS) & Health Multi Terminology Portal (HMTP)

A generic model was designed for the CISMeF_IS in order to fit all the terminologies into one global structure. This model was established around the "Descriptor" which is the central concept of the terminologies (aka "keyword"). The HMTP is a "Terminological Portal" connected to the CISMeF_IS to search terms among all the health terminologies available in French (or in English and translated into French) included in the CISMeF_IS and to search it dynamically. The ultimate goal is to use this search via the HMTP in order to:

• index manually or automatically resources in the CISMeF quality-controlled health gateway;

• allow a multi-terminology information retrieval [2];

It can also be very useful for teaching or performing audits in terminology management. Currently, the CISMeF_IS include 27 terminologies and classification, and therefore are user-accessible via the HMTP. Some terminologies and classifications are included in the UMLS meta-thesaurus (n = 9) but the majority are not (n = 18) such as ORPHANET [24], DRC [25], IUPAC [26]. Table 1 lists all the terminologies that are included in CISMeF_IS.

### The Foundational Model of Anatomy (FMA)

The FMA is an evolving formal ontology that has been under development at the University of Washington since 1994 [27,28]. It is the most complete ontology of human "canonical" anatomy. The FMA describes anatomical entities, most of which are anatomical structures composed of many interconnected parts in a complex way. Its objective is to conceptualize the physical objects and spaces that constitute the human body. It contains more than 81,000 classes and 139 relationships connecting the classes, and over 120,000 terms (preferred and synonyms) with 81,020 unique English preferred terms (PT)(Each FMA concept is represented by one preferred term and a list of synonyms), 52,040 unique English synonyms, 4,436 unique French terms and 139 French synonyms. An OWL-2 version of the FMA was recently proposed [29].

### French terminologies in anatomy

There are two standards for French anatomical terms [30]:

• an older one, Nomina Anatomica (NA) [31] published by the International Federation of Associations of anatomy in 1955;

• a more recent one which is the translation of the Terminologica Anatomica (TA) [30].

### Existing FMA

Out of 4,436 French PT included in the FMA, 1,110 of them were manually reviewed by a French anatomist (JM). This expert has proposed to modify the French label in 104 cases (9.5%) including two (2) mistakes. These modifications were mainly due to the fact that old NA was used instead of the TA. In these cases, the TA was chosen as the terminology for the PT instead of the NA, *e.g*. for the translation of the English term "ulna", the preferred term may come from TA as "ulna", and the synonym from NA as "cubitus". Adding these synonyms allows other health professionals to refer to the PT "ulna" when entering "cubitus" in the HMTP. The FMA was integrated into the HMTP since one year and is already available with a restricted access(http://pts.chu-rouen.fr/index.html?lang=en (click on "Connection"; login=fmauser and password=fmapass)). Since the integration of the FMA into the HMTP, 6,408 terms were manually translated into French (plus the original 4,436 French translations; +140%). The FMA was integrated into the UMLS in 2008 [32]. In UMLS the FMA is known as the University of Washington Digital Anatomist (UWDA) vocabulary. The UWDA consists of the Anatomy taxonomy (At) and selected structural relationships (part-of, branch-of, tributary of) [33].

## Methods

We compared two types of approaches to translate the FMA terms into French. The first one is UMLS-based on the conceptual information of the UMLS Metathesaurus. The second method is lexically-based on several Natural Language Processing (NLP) tools.

### UMLS-based Approach

**Figure 1:**
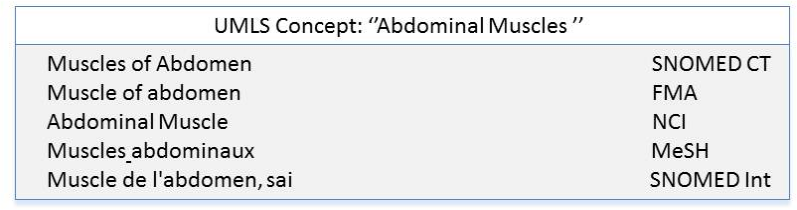
**translation of the FMA term "Muscle of abdomen" using the UMLS-Based approach**.

The first method relies on the UMLS Metathesaurus. It implies that each term to be translated must be included in the Metathesaurus. We use the six French terminologies included in the UMLS: the MeSH thesaurus, the SNOMED International (SNMI), the MedDRA, the ICD10, the WHO-ICPC2 and the WHO-ART. The French version of SNMI is not integrated into the UMLS Metathesaurus. However, its terms are tagged with UMLS CUIs, which permits integrating them into the Metathesaurus. The number of preferred terms from each terminology in the UMLS are reported in Table 2. The principle of the method is based on the conceptual construction of the UMLS Metathesaurus. For each FMA term in English included in the UMLS, we have extracted all UMLS concepts it belongs to. The next stage consists of deriving the set of all French terms that are related to the given concept, for each UMLS concept extracted in the first stage, i.e. all French terms that have the same CUI. For example, the FMA code "9620", which corresponds to the English term "Muscle of abdomen", is related to the UMLS concept "C0000739" ("Abdominal Muscles"). Then, in the second stage, two French terms may be associated to the English term "Muscle of abdomen" according to the UMLS concepts "C0000739" (see Figure 1).

**Table 2:** Number of Preferred terms from each terminologies in UMLS

Terminologies	Number of Preferred terms in UMLS
SNMI	107,900

MeSH	25,587

MedDRA	18,483

ICD10	9,308

WHO-ART	1,560

ICPS2	423

### Lexical Approach

In this approach, FMA terms in English from all bilingual terminologies (English and French) were normalized and we applied an algorithm to find terms in target terminologies which were the most lexically similar. When a correspondence was found, the translation of the English target term was proposed as one possible translation of the FMA term. This algorithm was exploited in several previously reported studies to map external French and English terminologies to UMLS and HMTP [17,34,35]. In this method, we used some Natural Language Processing tools developed by the NLM^® ^[36]. They were designed to help users in analyzing and indexing natural language texts in the medical field in English [37,38].

**Figure 2:**
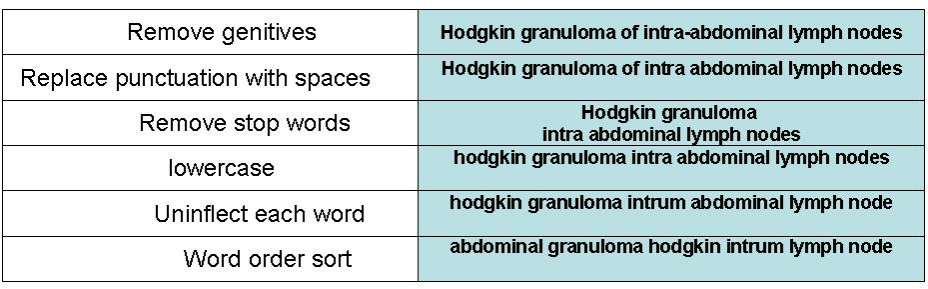
**Example of Normalization process for the FMA term "Hodgkin's granuloma of intra-abdominal lymph nodes"**.

We basically used the normalization program ("Norm") [39]: a program used to normalize English terminologies (UMLS terminology). The Normalization process involves stripping genitive marks, transforming plural forms into singular, replacing punctuation, removing stop words, lower-casing each word, breaking a string into its constituent words, and sorting the words into alphabetic order. In Figure 2 one can find a list of all stages to normalize the FMA term "Hodgkin's granuloma of intra-abdominal lymph nodes". Mapping used by this approach may provide three types of correspondences between all terms:

• Exact correspondence: if all the words that compose the two terms are exactly the same.

• Single to multiple correspondence: when the source term cannot be mapped by one exact target term, but can be expressed by a combination of two or more terms.

• Partial correspondence: in this type of mapping only a part of the source term will be mapped to one or more target terms.

In Table 3 one can find some examples of these three types of correspondences. In this work, only the exact and the single to multiple correspondences were studied. For example, based on this approach, the FMA term "Thymic branches of internal thoracic artery" is normalized into "artery branch internal thoracic thymic" which is mapped to the SNOMED International term "Thymic branches of internal thoracic artery". Finally, the corresponding French SNOMED International term "Rameaux thymiques de l'artère thoracique interne" was subsequently proposed as a possible translation of the English FMA term "Thymic branches of internal thoracic artery".

**Table 3:** Examples of the three types of mappings using lexical approach

Type of correspondence	FMA term	**French Term(s) **(English term)
Exact	Hand muscle	Muscle de la main (Muscles of hand)

Single to Multiple	Left dorsal scapular artery	Artère scapulaire postérieure (Dorsal scapular artery) and (+) Gauche (Left)

Partial	Abdominal extraperitoneal fascia	Fascia de l'abdomen (Fascia of abdomen, nos)

### Evaluation

#### Quantitative Evaluation

We investigated the coverage of the two methods according to the number of FMA PT translated into French. We also examined the coverage of the translated FMA terms by considering the French terminologies and the terms from these terminologies in the UMLS Metathesaurus and in CISMeF_IS for the lexically-based approach. We also compared the two approaches (lexical approach limited to the exact correspondence), by examining the number of different FMA PT translated by each approach. For each approach, we calculated the number of the English PT with at least one French translation (from the whole FMA, N = 81,020) and we calculated only the number of translations performed from FMA PT without French terms.

#### Qualitative Evaluation

The qualitative evaluation was performed on 100 translations from each approach by an expert on anatomy (OP). The samples of 100 FMA terms were randomly chosen. The expert was blinded to the two methods. This evaluation is clearly subjective using a six-levels scale for rating their quality: (a) "Very Good": the French translation corresponds exactly to the English FMA term; (b) "Good": the French translation is very good with no impact to the meaning but there are some minor differences such as missing punctuations like "-". For example, the translation of the FMA term "plantar tarsometatarsal ligaments" to the French term "ligaments tarsométatarsiens plantaires" was rated as "Good" because the anatomist considered that the French term is missing a "-" between "tarso" and "métatarsiens"; (c) "Average": the French translation will be more accurate if we delete or add some terms or when a singular term (*resp*. plural) is translated to plural (resp. singular); (d) "Bad": the French translation is false, but the corresponding term shares some true terms or the translation corresponds to a part of the original term. For example, the translation of the FMA term "tunica intima" to the French term "tunica intima" was rated as "Bad" because the term is "intima"; (e) "Very Bad": the French translation is false; (f) "cannot say": when the anatomist cannot evaluate the translation, when he does not know the FMA term for example. Examples of each evaluation are listed in Table 4.

**Table 4:** Examples of each type of evaluation used in this study

English	French	Evaluation
Thymic branches of internal thoracic artery	Rameaux thymiques de l'artère thoracique interne	Very Good

Plantar tarsometatarsal ligaments	Ligaments tarsométatersiens plantaires	Good

Interosseous metacarpal ligament	Ligaments métacarpiens interosseux	Average

Metatrsal interosseous ligaments	Ligaments intermétatarsiens interosseux	Bad

Blood capillary	Sang capillaire	Very Bad

Cell membrane protein	Protéines membranaires	CNS

## Results

### UMLS-based Approach

Using the UMLS-based approach, a set of 7,469 (9%) FMA PT were translated to at least one French term and 3,661 (49% of the 7,469) of them only exists in English in the FMA. Table 5 lists the number of French terms from each terminology proposed as a possible translation.

**Table 5:** Number of Terms from each terminology mapped to at least one term from FMA using the Knowledge Based approach

Terminology	Number of terms
SNOMED International	6,472

MeSH	1,419

MedDRA	9

WHO-ART	6

ICD10	5

For example, the FMA term "Abdomen" was translated to three possible French terms: "abdomen, sai", "abdomen" and "ventre".

### Lexical Approach

According to the exact type of mapping, 6,246 (7,7%) FMA PT were translated to at least one French term and 3,129 (50% of the 6,246) of them exist in English in the FMA. Table 6 lists the number of French terms from each terminology proposed as a possible translation.

**Table 6:** Number of Terms from each terminology mapped to at least one term from FMA using the Lexical Based approach

Terminology	Number of terms
SNOMED International	5,287

MeSH	1,340

ICD10	170

WHO-ICF	79

ATC	61

IUPAC	56

MedDRA	45

ORPHANET	31

MEDLINEPlus	24

WHO-ART	3

According to the "single to multiple" type of mapping, 27,761 FMA PT were translated to at least a combination of two French terms. However, these translations are not exact and need to be adjusted manually to construct the exact French term from the proposed one. For example, the FMA term "left third costotransverse foramen" was mapped to the three French terms: "trou de conjugaison postérieur de cruveilhier" (costotransverse foramen), "gauche"(left) and "troisième" (third). Using these three French terms a new term was constructed manually: "troisième trou de conjugaison postérieur gauche de cruveilhier", and was proposed as a possible translation of "left third costotransverse foramen".

**Figure 3:**
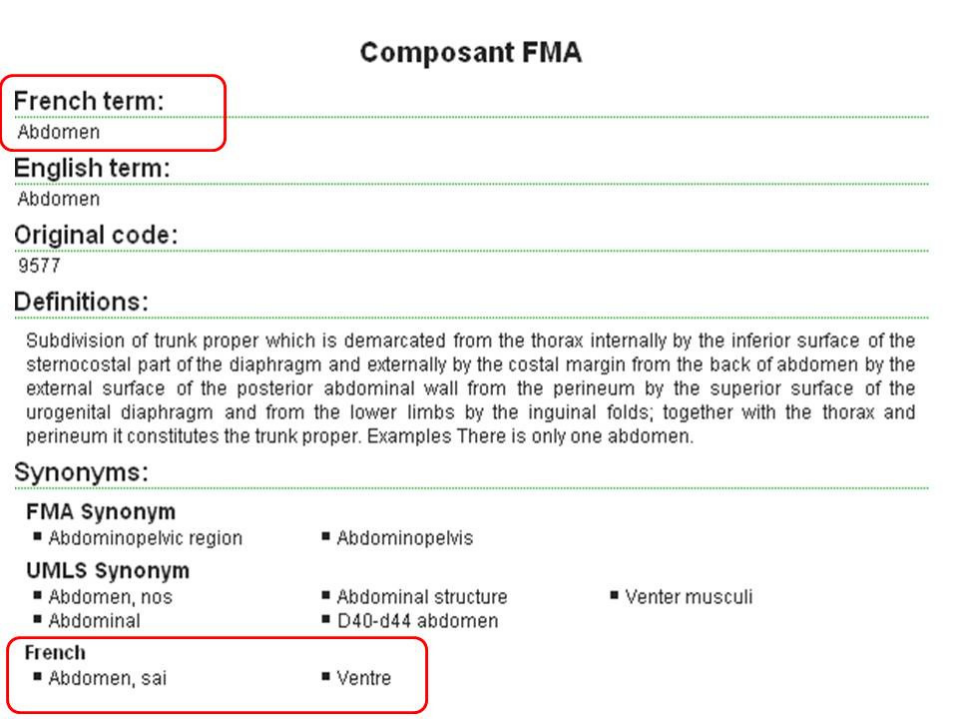
**FMA term "Abdomen" translated to three French terms: one chosen and two added as French synonyms**.

For the two approaches, more than one French term was proposed. However, only one term was manually chosen to be the unique translation of the English FMA term, the rest of terms were added as UMLS synonyms to the FMA term (or French synonyms in the case of the lexical approach if terms correspond to the valid mapping terms but not to the valid translation of the FMA PT). For example, for the FMA term "abdomen" the French term "abdomen" was chosen to be the French translation, whereas, the two terms "abdomen, sai" and "ventre" were added as UMLS synonyms (or French synonyms)(see Figure 3).

### Comparing the two approaches (UMLS-based Approach vs. Exact lexical approach)

#### Quantitative Comparison

**Figure 4:**
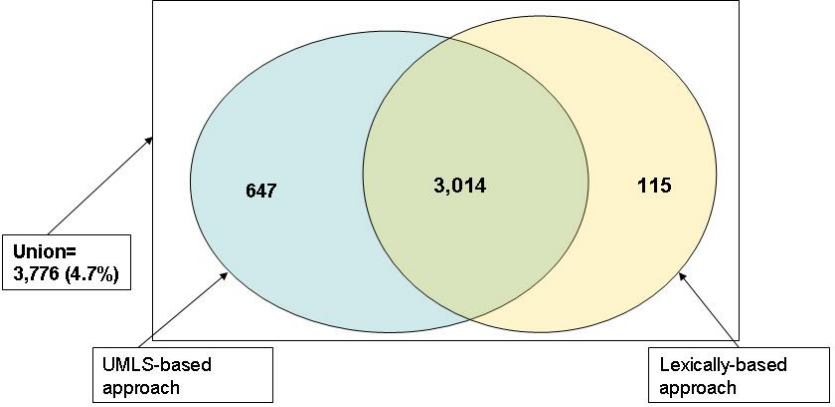
**The repartition of the number of FMA English terms translated by lexically and UMLS-based approaches**.

Using the UMLS-based approach 3,661 English FMA PT were translated when 3,129 FMA terms were translated by the exact lexical-based approach. From the FMA PT terms translated by the UMLS-based method, 647 terms are not in the set of those translated by the exact lexical methods and inversely, 115 FMA PT translated by the exact lexical method are not in the set of those translated by the the UMLS-based method (see Figure 4). When comparing Tables 5 and 6, only five terminologies were used by both methodologies: SNOMED International and MeSH provided more mapping by the UMLS-based approach than the lexical based approach respectively (6,472 *vs*. 1,419 and 5,287 *vs*. 1,340), whereas ICD10 provided more mapping for the lexical approach (170 *vs*. 5). WHO-ART and MedDRA provided very few mappings with both approaches (see Table 5 and Table 6).

#### Qualitative comparison

For the UMLS-based approach, 52 translations of the 100 submitted to an expert were rated as "very good" and only seven translations were rated as "bad" or "very bad ". For the lexical approach, 47 translations of the 100 submitted an expert were rated as "very good" and 20 were rated as "bad" or "very bad" (see Table 7). There is a significant difference between the two approaches (X^2 ^test, p = 0.015).

**Table 7:** Evaluation of 100 translations produced by the two approaches

	Very good	Good	Average	Bad	Very bad	NSP
**UMLS-based approach**	52	17	22	6	1	2

**Lexical approach**	47	10	15	13	7	8

There are two types of errors using the two approaches: (a) partial errors: only a part of the French term was correct but not entierly; (b) complete errors: all the words that compose the French term were false. In several cases, the expert proposed the right translation whereas the French term proposed was false (see Table 8).

**Table 8:** Examples of French translations proposed by expert

Approach	FMA term	French Term (false)	Proposition of expert
**UMLS-based approach **	intervertebral joint	articulation de la colonne vertébrale	articulation inter- vertébrale

**Lexical approach **	blood capillary	sang capillaire	capillaire

## Discussion

The aim of this study was to compare two approaches to translate a part of FMA vocabulary from English to French. The overall translation yield is quite low. Nevertheless, it has saved long hours for translators. The UMLS-based approach is straightforward and easy to implement. This approach has the advantage of domain knowledge included in the UMLS. In spite of the small number of French terminologies used from UMLS, the UMLS-based approach allows to acquire good quality translations. Qualitative evaluation demonstrated that 69% of the translations were rated as "very good" or "good".

On the other hand, the lexical approach is more difficult to implement but has the advantage of the large number of French medical terminologies included in CISMeF_IS. Qualitative evaluation demonstrate that 59% of the translations were rated as "very good" or "good". However, there are more translations rated as "bad" or "very bad" compared to the UMLS-based approach. The major types of translations rated as "bad" or "very bad" can be explained by three major reasons:

• translation from the singular to plural or *vice versa*. For example, the French translation "ligaments dorsaux du tarse" corresponds to the plural term of the exact French translation of the English FMA term: "dorsal tarsal ligament".

• the French translation is very specific or very broad compared to the FMA English term. For example, the French translation "deuxième facette métatarsien du premier cunéiforme" is broader than the English FMA term "second metatarsal facet of medial cuneiform bone". In this case, the expert proposed the exact French translation "deuxième facette métatarsiene du l'os cunéiforme médial".

• the French translation in the original terminology is not good. For example, the French translation "ligaments intermétatarsiens interosseux" of the FMA English term "metatarsal interosseous ligaments" is rated as false, however, this translation already exists between the two SNOMED Int. English and French terms. In this case, the expert proposed the French translation "ligaments métatarsiens interosseux" adapted to the FMA English term.

Using the two approaches, mapping between terms of different languages might vary in coverage depending on the terminology to be translated and on the target language. In our previously reported study [8] we translated a large number of MEDLINEPlus vocabulary in French due to the small number of terms. However, the small number of FMA terms automatically translated was not only due to the large number of terms but also to the limited anatomical coverage in all the terminologies used (*e.g*. ICD10, MedDRA, WHO-ART, ... etc.). The significant differences of mapping size by three terminologies (SNOMED International, MeSH, ICD10) used by the methods could be explained by the fact that ICD10 terms are based on the old NA where MeSH terms are based on the new TA and SNOMED International includes both of them. Although the use of lexical methods in the second produces high-quality alignments [40-43], the validity of the resulting lexical mappings is not guaranteed [44]. For example, in [45] the results of applying lexical mapping alone (using Metamap) with 70% coverage, 28.4% recall, 14.7% precision and almost 3 mappings per term. Therefore, the precision and recall was too low, and the ambiguity (3 mappings per term) high. Currently, structure-based techniques, which use structural properties like shared relationships across sources to find alignments between concepts, are applied in combination with lexical techniques, as it has been demonstrated that it increases the overall performance [42-44,46,47].

In contrast, the use of some approaches such as a corpus-based approach [12-16] or a statistical-based approach can offer more adapted and accurate translations. However, these approaches are limited in term of such parallel corpora and in term of low quantity of acquired translations. Nevertheless, a word by word translation [6] of terms may be a possible complementary approach.

Each approach can be improved: UMLS-based approach could benefit from additional French terminologies added to the UMLS Metathesaurus or more integrated terminologies translated in French. Due to this problem we proposed multiple approaches to map several French terminologies not integrated in the UMLS to the UMLS such as the Classification Commune des Actes Médicaux (CCAM)"A French coding system of surgical procedures" [17] and the ORPHANET database of rare diseases [35]. For the lexical approach, several improvements can be proposed to resolve problems due to the management of ambiguous acronyms across terminologies (*e.g*. CMT in MeSH ("Thyroid neoplasms" or "Charcot-marie-tooth disease")), or for the terms lexically close but with a different meaning, such as sterile as a "aspetic technique" and sterility as "Infertility". These two problems can be solved by using the UMLS semantic groups (SGs) [48] when the two terms are in the UMLS. Thus, mappings between two terms that do not share the same SGs would be filtered out. Another advantage of using the UMLS SGs is that it is easy to detect possible errors of translations between English terms and French terms from UMLS.

It has a real impact for a daily use of the FMA that could play a central role in French education and resources indexing. Since the main thesaurus such as MeSH lack precise anatomical terms, the FMA integration and translation in the CISMeF_IS is a great opportunity to improve the level of indexing to allow users querying very precise terms and subsequently to find relevant resources. The FMA translation will also improve the French translation of SNOMED CT and National Institute Common Terminology.

## Conclusions

In this paper, we present a methodology to translate the lexicon of the FMA ontology into French. We compare two approaches, a UMLS-based approach and a lexical approach. Overall, a low rate of translations were demonstrated by the two methods. Nevertheless, the two approaches permitted us to semi-automatically translate 3,776 FMA terms from English into French, this was to added to the existing 10,844 French FMA terms in the HMTP (4,436 FMA French terms and 6,408 FMA terms manually translated).

## Competing interests

The authors declare that they have no competing interests.

## Authors' contributions

TM, JG, LFS and SJD designed and developed the study and wrote the manuscript. OP evaluated the results of this study and JMM translated manually multiple English FMA to French and continue to help us to translate and validate the rest of FMA. All authors read and approved the final manuscript.

## Pre-publication history

The pre-publication history for this paper can be accessed here:

http://www.biomedcentral.com/1472-6947/11/65/prepub
